# *QuickStats:* Percentage* of Children and Adolescents Aged 3–17 Years Who Ever Received a Diagnosis of Autism Spectrum Disorder,^†^ by Family Income,^§^ 2020–2022

**DOI:** 10.15585/mmwr.mm7315a5

**Published:** 2024-04-18

**Figure:**
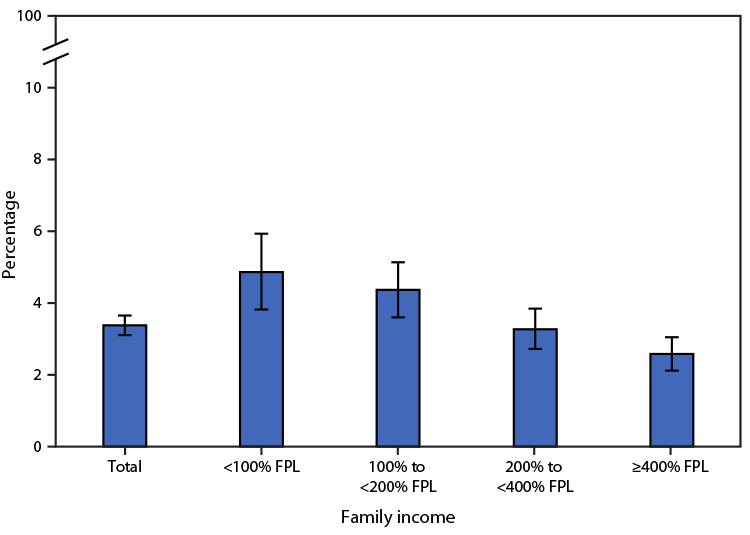
During 2020–2022, 3.4% of children and adolescents aged 3–17 years had received a diagnosis of autism spectrum disorder. The prevalence of autism spectrum disorder among children and adolescents decreased as family income increased.

**Supplementary** **Table:** https://stacks.cdc.gov/view/cdc/152917

**Source:** National Center for Health Statistics, National Health Interview Survey, 2020–2022. https://www.cdc.gov/nchs/nhis.htm

For more information on this topic, CDC recommends the following link: https://www.cdc.gov/ncbddd/autism/index.html

